# Supplementary material for: Metabolome and Transcriptome Analyses Unravel the Molecular Regulatory Mechanisms Involved in Photosynthesis of Cyclocarya paliurus under Salt Stress
Source: Int J Mol Sci. 2022 Jan 21;23(3):1161. doi: 10.3390/ijms23031161 (PMC8835658; doi:10.3390/ijms23031161)
Supplement: Supplementary file 1 [file ijms-23-01161-s001.zip › ijms-1554687supplementary materials.pdf]

International Journal of *Molecular Sciences*

Article

Metabolome and Transcriptome Analyses Unravel the Molecular Regulatory Mechanisms Involved in Photosynthesis of *Cyclocarya paliurus* under Salt Stress

Lei Zhang [1], Zijie Zhang [1], Shengzuo Fang [1,2,*], Yang Liu [1] and Xulan Shang [1,2]

[1] College of Forestry, Nanjing Forestry University, Nanjing 210037, China; zhanglei321@njfu.edu.cn (L.Z.); iszhangzj@sina.com (Z.Z.); lyang_188@sina.com (Y.L.); shangxulan@njfu.edu.cn (X.S.)
[2] Co-Innovation Centre for Sustainable Forestry in Southern China, Nanjing Forestry University, Nanjing 210037, China
* Correspondence: fangsz@njfu.edu.cn or fangsz@njfu.com.cn, Tel.: +86-25-854-28603

Citation: Zhang, L.; Zhang, Z.; Fang, S.; Liu, Y.; Shang, X. Metabolome and Transcriptome Analyses Unravel the Molecular Regulatory Mechanisms Involved in Photosynthesis of *Cyclocarya paliurus* under Salt Stress. *Int. J. Mol. Sci.* **2022**, *23*, 1161. https://doi.org/10.3390/ijms23031161.

Academic Editors: Vitaliy Borisov

Received: 29 December 2021
Accepted: 17 January 2022
Published: 21 January 2022

Publisher's Note: MDPI stays neutral with regard to jurisdictional claims in published maps and institutional affiliations.

Copyright: © 2022 by the authors. Licensee MDPI, Basel, Switzerland. This article is an open access article distributed under the terms and conditions of the Creative Commons Attribution (CC BY) license (https://creativecommons.org/licenses/by/4.0/).

Table S1. Total carbon and total nitrogen contents of *C. paliurus* leaves under different salt treatments.

Sampling Time	Treatment	Nitrogen (%)	Carbon (%)
T_1	CK	2.60 ± 0.08 Aa	45.65 ± 0.09 Ba
	LS	2.22 ± 0.07 Bb	45.20 ± 0.05 Bab
	MS	2.20 ± 0.07 Bb	44.96 ± 0.24 Ab
	HS	2.19 ± 0.12 Ab	43.66 ± 0.42 Ac
T_2	CK	2.92 ± 0.29 Aa	48.67 ± 0.41 Aa
	LS	2.81 ± 0.28 Aa	46.99 ± 0.18 Ab
	MS	2.48 ± 0.15 Aa	45.12 ± 0.69 Ac
	HS	1.96 ± 0.17 Ab	43.20 ± 0.60 Ad

Different capital letters indicate a significant difference ($p < 0.05$) between two sampling times under the same salt concentration. Different small letters indicate a significant difference ($p < 0.05$) among different salt treatments at each sampling time. Refer to Figure 1 for CK, LS, MS, HS, T_1 and T_2.

Table S2. Difference analysis of metabolite abundance.

Metabolites	Log2_FC					
	T_1			T_2		
	LS vs CK	MS vs CK	HS vs CK	LS vs CK	MS vs CK	HS vs CK
2-(alpha-Hydroxyethyl) thiamine diphosphate	0.89	0.52	1.28	0.22	0.21	−3.55 *
2-Deoxy-D-ribose 1-phosphate	0.45	0.11	−0.18	−0.14	−0.03	0.03
3-Phospho-D-glycerate	−0.29	0.00	0.18	−0.29	−0.81	0.10
3-Phospho-D-glyceroyl phosphate	2.58	3.15	2.36	−0.32	−0.09	3.01
5-Phospho-alpha-D-ribose 1-diphosphate	−0.54	0.09	0.34	−0.26	1.36 *	0.07
Acetyl-CoA	0.02	−0.57	0.37	0.42	−1.04*	0.15
Arbutin	0.08	0.02	0.07	−0.11	−0.60 *	0.11
Arbutin 6-phosphate	0.90 *	1.92 *	2.11 *	−0.34	−1.90 *	1.86 *
Beta-D-Glucose	−0.47 *	−0.37	−0.05	0.12	0.04	-0.43
cis-Aconitate	−0.81	0.05	−1.65 *	2.09*	4.24 *	0.96 *
Citric acid	1.02	0.20	0.05	0.41	0.81	0.10
D-Fructose 1,6-bisphosphate	1.09 *	2.05*	1.72 *	0.51	1.88 *	0.97 *
D-Glucono-1,5-lactone 6-phosphate	0.58 *	−0.06	0.63 *	0.22	−0.19	2.07 *
D-Ribose 5-phosphate	0.30	−0.08	-0.05	−0.08	−0.29	−0.27
Fructose 6-phosphate	0.26 *	0.03	−0.18	−0.19	0.06	−0.80 *
Fumaric acid	−1.23 *	−1.70 *	−0.51 *	−1.75*	−7.24 *	−4.90 *
Gluconic acid	−1.32	−1.18	−0.87	−0.11	−1.28 *	0.99
Gluconolactone	0.55 *	0.72	0.80	−0.20	−0.06	−0.31
Glyceric acid	−1.03 *	−1.47 *	−0.83 *	−0.30	−1.50 *	−0.44
Glycerone phosphate	0.46	0.81	0.37	-0.63	-0.94 *	0.05
Glycogen	0.16	−1.09 *	−0.82	1.98*	1.29 *	0.10
Isocitric acid	0.07	−0.70	0.49	−1.27*	−3.62 *	−2.43 *
L-Malic acid	−1.14	−1.81	−0.99	−1.69*	−4.18 *	−3.35
Oxaloacetate	1.06 *	1.06 *	1.06 *	−0.13	−0.53 *	−0.19
Phosphoenolpyruvate	0.09	−1.41 *	−0.34	−2.38*	−3.43 *	1.14
Pyruvic acid	1.07	0.06	1.16	−1.14*	−1.51 *	−1.05
Salicin	-0.12	0.88	0.42	3.86*	4.31 *	3.22 *
Salicin 6-phosphate	0.10	0.81 *	0.73	−1.00*	−0.87 *	−1.01 *
Sedoheptulose	−0.51 *	−0.14	0.13	0.46	1.29*	0.08
Sedoheptulose 1,7-bisphosphate	−0.56 *	−0.31	−0.19	−0.30	−0.72	0.37 *
Sedoheptulose 7-phosphate	0.30	1.17 *	1.01 *	−0.33	0.93 *	−0.16 *
Succinic acid	−0.56 *	−0.85 *	−0.39 *	−0.23	−1.12 *	−0.73 *

* indicates significant differences ($p < 0.05$). Refer to Figure 1 for CK, LS, MS, HS, T_1 and T_2.

Table S3. Identification of photosynthetic genes.

Gene_ID	Symbol	Description
Unigene0004807	FDX3	Ferredoxin, root R-B2-like [*Juglans regia*]
Unigene0008364	PETH	Ferredoxin--NADP reductase, leaf-type isozyme, chloroplastic [*Juglans regia*]
Unigene0017274	PSBR	Photosystem II 10 kDa polypeptide, chloroplastic [*Juglans regia*]
Unigene0018500	PNSL3	Photosynthetic NDH subunit of lumenal location 3, chloroplastic [*Juglans regia*]
Unigene0019049	AP1	Ferredoxin-like [*Juglans regia*]
Unigene0022449	PSAO	Photosystem I subunit O [*Juglans regia*]
Unigene0025814	PSAN	Photosystem I reaction center subunit N, chloroplastic [*Juglans regia*]
Unigene0031001	PSB28	Photosystem II reaction center PSB28 protein, chloroplastic [*Juglans regia*]
Unigene0033466	ATPG	ATP synthase subunit b', chloroplastic [*Juglans regia*]
Unigene0040136	PPL1	PsbP-like protein 1, chloroplastic [*Juglans regia*]
Unigene0042116	ATPC	ATP synthase gamma chain, chloroplastic-like [*Juglans regia*]
Unigene0043079	PSAEA	Photosystem I reaction center subunit IV, chloroplastic-like [*Juglans regia*]
Unigene0043080	PSAEA	Photosystem I reaction center subunit IV B, chloroplastic-like [*Juglans regia*]
Unigene0043925	PSBQ2	Oxygen-evolving enhancer protein 3-2, chloroplastic-like [*Juglans regia*]
Unigene0045582	PSBA	Photosystem II protein D (chloroplast) [*Ficus racemosa*]
Unigene0046028	PSBC	photosystem II 44 kDa protein (chloroplast) [*Carpinus putoensis*]
Unigene0049595	PSAF	Photosystem I reaction center subunit III, chloroplastic-like [*Juglans regia*]
Unigene0049596	PSAF	Photosystem I reaction center subunit III, chloroplastic-like [*Juglans regia*]
Unigene0050921	PSAA	Photosystem I P700 apoprotein A1 (chloroplast) [*Vicia sativa*]
Unigene0052289	PSBW	Photosystem II reaction center W protein, chloroplastic-like [*Juglans regia*]
Unigene0052565	ATPC	ATP synthase gamma chain, chloroplastic [*Juglans regia*]
Unigene0063197	PSAG	Photosystem I reaction center subunit V, chloroplastic [*Juglans regia*]
Unigene0066529	PSBP	Oxygen-evolving enhancer protein 2, chloroplastic [*Juglans regia*]
Unigene0066594	PSAC	Photosystem I subunit VII (chloroplast) [*Castanopsis concinna*]
Unigene0068142	PNSL1	Photosynthetic NDH subunit of lumenal location 1, chloroplastic [*Juglans regia*]
Unigene0070030	PETE	Plastocyanin B'/B'' [*Juglans regia*]
Unigene0071208	PSAL	Photosystem I reaction center subunit XI, chloroplastic [*Juglans regia*]
Unigene0074242	FDC1	Ferredoxin, root R-B2 [*Juglans regia*]
Unigene0076061	PSBS	Photosystem II 22 kDa protein, chloroplastic-like [*Musa acuminata*]
Unigene0081034	PSAD	Photosystem I reaction center subunit II, chloroplastic-like [*Juglans regia*]
Unigene0084750	FDC2	Ferredoxin [*Dorcoceras hygrometricum*]
Unigene0084871	PSBY	Photosystem II core complex proteins psbY, chloroplastic [*Juglans regia*]
Unigene0087934	FDX3	Ferredoxin, root R-B1-like [*Juglans regia*]
Unigene0088051	PSB27-1	Photosystem II repair protein PSB27-H1, chloroplastic [*Juglans regia*]
Unigene0088777	PSBS	Photosystem II 22 kDa protein, chloroplastic [*Juglans regia*]
Unigene0090288	PSBB	Photosystem II 47 kDa protein (chloroplast) [*Juglans regia*]
Unigene0100344	ATPD	ATP synthase subunit delta, chloroplastic-like [*Juglans regia*]
Unigene0100448	PSBO	Oxygen-evolving enhancer protein 1, chloroplastic [*Juglans regia*]
Unigene0100785	PSAK	Photosystem I reaction center subunit psaK, chloroplastic [*Prunus mume*]

Unigene0101657	PSAH	Photosystem I reaction center subunit VI, chloroplastic-like [*Juglans regia*]
Unigene0101658	PSAH2	Photosystem I reaction center subunit VI-2, chloroplastic [*Juglans regia*]
Unigene0102180	PSBS	Photosystem II 22 kDa protein, chloroplastic [*Juglans regia*]

Table S4. Identification of the genes encoding transcription factor.

Gene_ID	Symbol	Description
Unigene0072178	ORRM6	Glycine-rich RNA-binding protein 4, mitochondrial isoform X2 [*Juglans regia*]
Unigene0007735	NAC100	NAC domain-containing protein 100-like [*Juglans regia*]
Unigene0074345	ERF003	Ethylene-responsive transcription factor ERF003-like [*Juglans regia*]
Unigene0005122	ERF3	Ethylene-responsive transcription factor 3-like [*Juglans regia*]
Unigene0001734	BPC6	Protein BASIC PENTACYSTEINE6-like isoform X1 [*Juglans regia*]
Unigene0074995	MYR2	MYB-related protein 2-like [*Juglans regia*]
Unigene0099140	AG2	Floral homeotic protein AGAMOUS isoform X1 [*Juglans regia*]
Unigene0041578	RVE6	Protein REVEILLE 6 isoform X2 [*Juglans regia*]
Unigene0085912	ASIL2	Trihelix transcription factor ASIL1-like [*Juglans regia*]
Unigene0076114	MPS1	Serine/threonine-protein kinase mph1 isoform X1 [*Juglans regia*]
Unigene0020049	ABR1	Ethylene-responsive transcription factor ABR1-like [*Juglans regia*]
Unigene0048500	NAC017	NAC domain-containing protein 17-like [*Juglans regia*]
Unigene0054558	RVE8	Protein REVEILLE 8-like isoform X1 [*Juglans regia*]
Unigene0050441	DOF1.5	Dof zinc finger protein DOF1.5-like [*Juglans regia*]
Unigene0013496	IDD7	Protein indeterminate-domain 7-like isoform X2 [*Juglans regia*]
Unigene0011569	MYB3R5	MYB-related protein B-like [*Juglans regia*]
Unigene0034530	TCP20	Transcription factor TCP20-like [*Juglans regia*]
Unigene0042582	ZHD6	Zinc-finger homeodomain protein 6 [*Juglans regia*]
Unigene0053531	BZIP17	bZIP transcription factor 17-like [*Juglans regia*]
Unigene0025559	HSFB3	Heat stress transcription factor B-3-like [*Juglans regia*]
Unigene0088675	REF6	Lysine-specific demethylase REF6-like [*Juglans regia*]
Unigene0028145	ERF3	Ethylene-responsive transcription factor 3-like [*Juglans regia*]
Unigene0017097	ERF113	Ethylene-responsive transcription factor ERF113-like [*Juglans regia*]
Unigene0091789	ERF035	Ethylene-responsive transcription factor ERF039-like [*Juglans regia*]
Unigene0066890	RAP2-1	Ethylene-responsive transcription factor RAP2-1-like [*Juglans regia*]
Unigene0032570	DREB3	Dehydration-responsive element-binding protein 3-like [*Juglans regia*]
Unigene0014544	IDD14	Protein SHOOT GRAVITROPISM 5-like [*Juglans regia*]

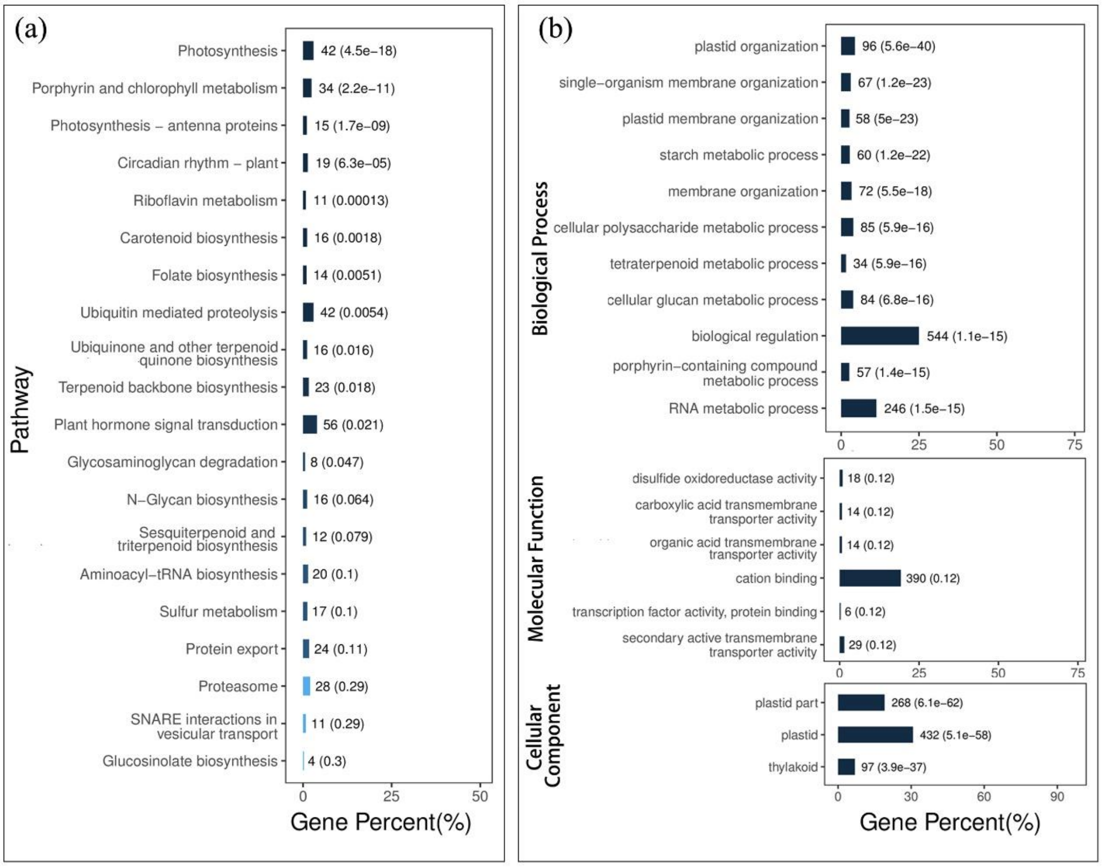

Figure S1. (**a**) Kyoto Encyclopedia of Genes and Genomics (KEGG) and (**b**) Gene Ontology (GO) enrichment analysis of DEGs in cyan module. Bracketed text indicates *q*-value.
